# Maintained Visual-, Auditory-, and Multisensory-Guided Associative Learning Functions in Children With Obsessive–Compulsive Disorder

**DOI:** 10.3389/fpsyt.2020.571053

**Published:** 2020-11-26

**Authors:** Ákos Pertich, Gabriella Eördegh, Laura Németh, Orsolya Hegedüs, Dorottya Öri, András Puszta, Péter Nagy, Szabolcs Kéri, Attila Nagy

**Affiliations:** ^1^Department of Physiology, Faculty of Medicine, University of Szeged, Szeged, Hungary; ^2^Faculty of Health Sciences and Social Studies, University of Szeged, Szeged, Hungary; ^3^Vadaskert Child and Adolescent Psychiatric Clinic, Budapest, Hungary

**Keywords:** basal ganglia, hippocampus, equivalence learning, generalization, psychophysics, pediatric

## Abstract

Sensory-guided acquired equivalence learning, a specific kind of non-verbal associative learning, is associated with the frontal cortex–basal ganglia loops and hippocampi, which seem to be involved in the pathogenesis of obsessive–compulsive disorder (OCD). In this study, we asked whether visual-, auditory-, and multisensory-guided associative acquired equivalence learning is affected in children with OCD. The first part of the applied learning paradigm investigated association building between two different sensory stimuli (where feedback was given about the correctness of the choices), a task that critically depends upon the basal ganglia. During the test phases, which primarily depended upon the hippocampi, the earlier learned and hitherto not shown but predictable associations were asked about without feedback. This study involved 31 children diagnosed with OCD according to the *Diagnostic and Statistical Manual of Mental Disorders, 5th Edition* (*DSM-V*) criteria and 31 matched healthy control participants. The children suffering from OCD had the same performance as the control children in all phases of the applied visual-, auditory-, and multisensory-guided associative learning paradigms. Thus, both the acquisition and test phases were not negatively affected by OCD. The reaction times did not differ between the two groups, and the applied medication had no effect on the performances of the OCD patients. Our results support the findings that the structural changes of basal ganglia and hippocampi detected in adult OCD patients are not as pronounced in children, which could be the explanation of the maintained associative equivalence learning functions in children suffering from OCD.

## Introduction

Obsessive–compulsive disorder (OCD) is one of the most prevalent human psychiatric disorders, affecting 2–3% of the adult population, and its prevalence is the same in children and adolescents (1). Several types of obsessions can develop as a result of OCD, and several compulsions will emerge to mitigate or weaken the obsessions (2). Several neurobiological abnormalities can be found in these patients, which can be observed in both morphological and functional alterations in comparison with healthy controls. Structural and functional imaging of OCD patients has revealed higher cortical activation in the limbic and frontal associative cortices and the connected deep brain structures in the basal ganglia [for a review, see (3, 4)] and hippocampi. Thus, the cortico-basal ganglia-cortical loops and hippocampi seem to be strongly involved in the pathogenesis of OCD (5).

Several studies have addressed the cognitive functions of OCD patients. Concerning memory and learning functions, the results of these studies have not been uniform. Both verbal and non-verbal memory functions had alterations in some studies and did not in others (for a review, see (6) in adults and (7) in children). However, to our knowledge, there is no information about a specific kind of the non-verbal learning of acquisition acquiring and the connected memory processes in pediatric OCD patients. Acquired equivalence learning, a specific kind of non-verbal associative learning, is connected to the above-mentioned frontal cortex–basal ganglia loops and hippocampi but has not yet been investigated in children and adolescents with OCD. The Rutgers Acquired Equivalence Test developed by Myers et al. (8) is a commonly applied test to investigate this learning function (9–14) A great advantage of this test is that each phase of the paradigm has well-described neural substrates. It has two main phases, the acquisition phase, which depends primarily upon the function of the frontal cortex–basal ganglia loop (8, 12), and the test phase, which depends primarily upon the hippocampi and the mediotemporal lobe (8, 15). The basal ganglia and the hippocampi are both brain structures that are fundamentally involved in visual associative learning, and they are multisensory structures of the mammalian brain (16–19). Although the original acquired equivalence learning test only applies visual stimuli, we developed and validated an auditory- and multisensory (audiovisual)-guided equivalence learning paradigm with the same structure as the original visual one (20) to investigate multisensory-guided acquired equivalence learning. OCD is primarily connected to the dysfunction of the frontal cortex–basal ganglia loops; thus, we hypothesized that the acquisition phases would primarily be affected during the applied acquired equivalence tests. Thus, the primary aim of the present study was to determine whether visual-, auditory-, and multisensory-guided associative acquired equivalence learning and the connected memory processes (retrieval and generalization) would be affected in children with OCD. The main motivation of the present study was that in OCD, especially in children and adolescents, the studies about non-verbal learning are underrepresented. We are positive that the clarifying of these issues could contribute to the understanding of behavioral changes in OCD patients and could possibly give several useful insights to applicable new behavioral therapies for patients suffering from OCD.

## Materials and Methods

### Subjects

During data collection, 43 pediatric OCD patients from the Vadaskert Child and Adolescent Hospital (Budapest, Hungary) were involved in the research. We had to exclude 12 of them from the further analysis because of the occurrence of several comorbidities beside the OCD. Four of them had attention deficit hyperactivity disorder, four had autism spectrum disorder, three had some mood disorder, and one of them had epilepsy. This study included 31 pediatric OCD patients without any comorbidity (*n*_male_ = 18), aged 7.5–17.5 (mean, 12.63 ± 2.72). All participants were White, free of ophthalmological, otological, neurological, or psychiatric conditions besides OCD. The diagnosis of OCD was made by board-certified child psychiatrists according to the *Diagnostic and Statistical Manual of Mental Disorders, 5th Edition* (*DSM-V*) manual. To exclude disorders of color vision, the Isihara plate assessment was used prior to testing (21). We estimated participants' IQ levels using the Colored Raven Progressive Matrices (22–24). Of the 31 children diagnosed with OCD, 16 were being treated with medications at the time of the tests (medicated group), while the other 15 were medication free during and before the investigation (unmedicated group). Fifteen children with OCD were receiving selective serotonin reuptake inhibitors (SSRIs, such as fluvoxamine, sertraline, or escitalopram), and one was receiving SSRI + SNRI (selective serotonin reuptake inhibitor and selective norepinephrine reuptake inhibitor: clomipramine). Three of the patients medicated with SSRI received other medications as well (clomipramine, benzodiazepine, or an atypical antipsychotic: risperidone).

The control group included children without any known psychiatric, neurological, or neurodevelopmental disorders. The controls were White, free of ophthalmological and otological problems. From our database of control children recruited from local schools, 31 control children (*n*_male_ = 18; mean age, 12.63 ± 2.73 years; range, 7.5–17.5 years) were assorted, who were matched one-to-one based on sex, age (differing in age by no more than 6 months), and IQ level to the patient group. The IQ level of the control children was estimated similarly to the OCD patients with the Standard and Colored Raven Progressive Matrices (22–24).

The children and their parents were informed about the background and goals of the study as well as the procedures involved. It was also emphasized that the participants were free to quit the study at any time (no one did so), and no compensation or benefit was given at the end of the tasks. Each participant and parent signed an informed consent form. The protocol of the study conformed to the tenets of the declaration of Helsinki in all respects, and it was approved by the Ministry of Human Capacities, Budapest, Hungary (11818-6/2017/EÜIG).

### Learning Paradigms

The tests were run on a personal computer, with the visual stimuli presented on a cathode-ray tube (CRT) screen and the auditory stimuli on a Sennheiser HD 439 using closed, over-ear headphones. The tests were conducted in a quiet room with the participants sitting at a standard distance (114 cm) from the screen. The M and X keys of the keyboard were labeled left and right, respectively. The children were asked to press the left key with the left hand, and the right key with the right hand. One child was tested at a time without a time limit to enable each participant to focus undividedly on the learning. No forced quick responses were expected; however, the response times were measured during the three phases of the test. The original visual associative learning test (8) that was written for iOS was slightly modified, translated into Hungarian, and rewritten in Assembly for Windows with the written permission of Professor Catherine E. Myers (Rutgers University, NJ, USA), the corresponding author of the previously cited article. Auditory- and multisensory (audiovisually)-guided tests were also introduced and implemented in Assembly for Windows by our group (20). During these tests, the children had to learn to associate two independent pieces of information, referred to as the antecedent and the consequent. The participants were asked to learn associations of antecedent and consequent stimuli through trial and error during the first half of the task and indicate their choice by pressing either the X (left) or M (right) button on the keyboard. The left or right button corresponded to a picture on the respective side of the screen when the visual stimuli were presented (visual and multisensory paradigms). All three paradigms consisted of two different parts: the acquisition and the test phases. During the first half, the acquisition phase, the participant had to form associations between the presented stimuli (equivalence acquisition), and the program gave feedback about the success of the current trial (a green check mark if the answer was correct or a red X if not). After a given number of correct answers, new pairs were introduced one by one until six of the eight possible pairs had been presented. The second test phase can be further divided into two parts: retrieval and generalization. During the retrieval part, the child had to recall the pairs associated in the acquisition phase, while during the generalization part, two new hitherto unknown pairs were presented that were predictable based on the previously seen ones. The test phase had a fixed number (48) of trials, 12 of which included new associations, which were mixed among the already known ones from the retrieval phase. As mentioned above, the children had to give a certain number of correct answers in a row after the presentation of each new pair before they were able to proceed to the next new pair or the second half of the test. The number of consecutive correct answers required was four after the presentation of the first two pairs, and the number increased by two after each new pair was introduced (thus, 12 correct answers in a row were required after all six pairs were introduced to progress to the second half of the test). This ensured that the children associated all the presented pairs and greatly diminished their chances of getting to the second phase based on pure luck. This also meant that the number of trials in the acquisition phase varied from participant to participant depending on their performance.

#### Visual Paradigm

**Figure 1:**
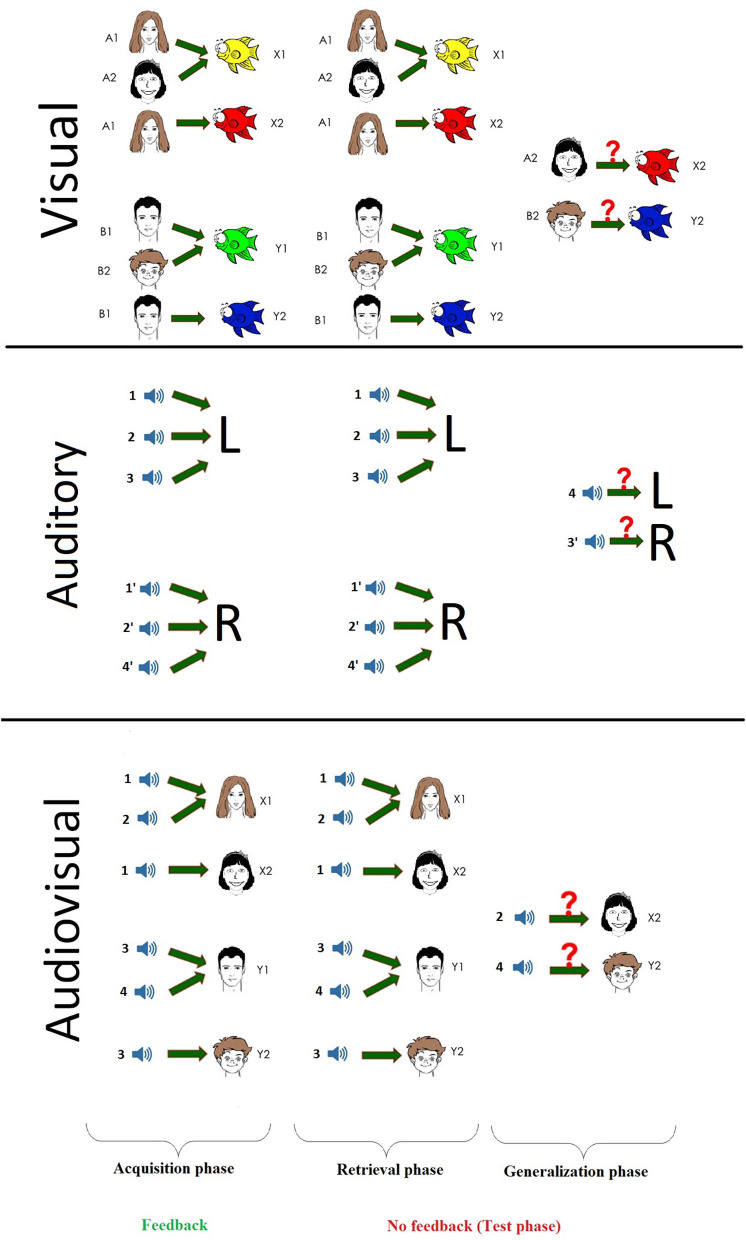
The schematic parts of the three different paradigms.

The visual paradigm was a slightly modified version of the Rutgers Acquired Equivalence Test (RAET) of (8). During any given trial, the participant was shown a drawn face in the upper middle part of the screen with two colored fish below the face and was asked to choose a fish. The possible faces were all cartoon-like drawings of a boy, girl, woman, or man. The four fish were of identical size and shape, differing only in color: yellow, green, red, or blue. The four faces (A1, A2, B1, and B2) and the four different colored fish (X1, X2, Y1, and Y2) could create eight possible pairs during a single session of the task. These combinations were based on the antecedents (the faces). At the start of the task, the program randomly chose whether the faces of the same sex, same age, or same color hair belonged together. At the beginning of the acquisition phase, the children had to learn that when face A1 or A2 was shown on the screen, it belonged with fish X1, not fish Y1 (green or yellow, respectively). The same applied to when face B1 or B2 was presented. The correct answer (fish) was Y1, not X1, the exact opposite of the previous scenario. This also meant that the children learned that faces A1 and A2 were equivalent in their consequents (belonged with the same fish, e.g., the yellow one) as faces B1 and B2 (belonged to the other fish, e.g., the green one). It clearly follows from the possible combinations that A1 and A2 could be the girl and the boy (same age), the girl and the woman (same sex), or the girl and the man (same hair color). Figure 1 shows only one possible iteration of the task. In the next stage of the acquisition phase, new consequents (red and blue fish) were introduced. If face A1 was shown, participants were expected to associate it with fish X2 (because the face was already associated with fish X1), not Y2, and in case of face B1, the correct answer was again the exact opposite (B1–Y2). When the aforementioned six pairs were presented and the participant gave 12 correct answers in a row, the test phase began. Up to this point, the children had received visual feedback in the form of a green check mark (correct answer) or red X (incorrect answer). During the test phase, no further feedback was given, and the program presented two new hitherto unknown combinations (faces A2 and B2 with the red and blue fish, X2 and Y2, respectively) mixed with the six already learned pairs. The participants had no knowledge of any possible new associations beforehand; however, if they learned that A1 and A2 were equivalent, similarly to B1 and B2, they could generalize about the previously learned associations and pair fish X2 with face A2 (the fish associated with A1) and fish Y2 with face B2 (the fish associated with face B1) (Figure 1, upper part).

#### Auditory Paradigm

In the auditory task, the participants had to learn to associate sounds (antecedents) with the left or right buttons (L or R as consequents in Figure 1, middle panel). Eight different sounds, distributed into four pairs, were used (1–4 and 1′-4′): a Hungarian word said by a male and a female voice in an emotionally neutral tone; the sound of two musical instruments, a piano and a guitar; the sound of two animals, a dog and a cat; and the sound of two vehicles, a motorcycle and the ignition of a car engine. The participants were required to listen to each sound in pairs once before the beginning of the task. The different sound pairs were introduced in a random manner during the task. For instance, during one iteration of the task, sounds 1 and 1′ were the animal sounds, while in another case, they were the human voices, and so on. Each sound was 1.5 s long and had the same intensity [sound pressure level (SPL) = 60 dB]. The grouping of sounds to pairs was reflected in the possible associations of sounds and buttons: If one half of the sound (e.g., 1) belonged to the left button (X), then the pair of sounds belonged to the right button (1′–M). The children were expected to associate the pairs through trial and error based upon the same visual feedback (green check mark or red X) as previously described in *Visual Paradigm*. During the acquisition phase, the participants learned to associate two pairs of sounds (1, 1′, 2, 2′) with the left and right buttons (four associations altogether), thus associating the pairs. Then, one sound from each of the two remaining groups (3, 4) was presented and learned, ending the acquisition phase with six of the eight possible sounds associated to buttons, similar to the visual paradigm. The test phase went in the exact same manner as described in detail in *Visual Paradigm*: 36 trials for the already learned associations and 12 for the two new sounds (3′, 4′) mixed together. Although all of the learning tasks contained eight stimuli, in the auditory paradigm, in contrast to the visual and multisensory test—in which two visual stimuli or an auditory stimulus and a visual stimulus had to be associated—the sound had to be associated not with a second sound but with a particular button (Figure 1, middle part).

#### Multisensory (Audiovisual) Paradigm

The experimental setting of the paradigm was very similar to the one used for the visual paradigm, with the nature of the stimuli being the only difference. Four clearly distinguishable sounds (one of each pair of the antecedents from the auditory paradigm: a female voice saying a Hungarian word in an emotionally neutral tone, a cat meowing, a musical tone played by a guitar, and the sound of a car's engine starting) were used as antecedents (s1, s2, s3, and s4), and four cartoon-like drawn faces were used as consequents (X1, X2, Y1, and Y2). During each trial, one of the described sounds was played on headphones (SPL = 60 dB), while two different faces were shown on the screen to the participant, who was asked to associate a face to the sound. During the acquisition phase, the children were expected to learn which sound went with which face with the help of visual feedback (green check mark or red X) after pressing the button corresponding to the side of the screen where the face was presented (X for left side and M for right side) (Figure 1, bottom part).

### Data Analysis

The number of trials, accuracy of responses, and response times were analyzed in three different groups for each of the paradigms: the acquisition phase, the retrieval, and the generalization parts of the test phase. The number of trials necessary to complete the acquisition phase [number of acquisition trials (NAT)], the number of correct and incorrect choices during the acquisition phase, and the number of correct and incorrect answers for known and unknown associations during the retrieval and generalization parts of the test phase were registered. From the aforementioned data, error ratios were calculated: the ratio of correct answers to all answers in the acquisition phase [acquisition learning error ratio (ALER)], in the retrieval part of the test phase [retrieval error ratio (RER)], and in the generalization part of the test phase [generalization error ratio (GER)]. Response times (RTs) were measured in milliseconds with microsecond accuracy for every answer in each phase. RTs exceeding the mean ± 3 SD of the participants' response times were excluded from further analysis. The order of the three different tasks (visual/auditory/multisensory) was varied randomly across participants to avoid a carryover effect among the paradigms.

After testing for normality distribution using the Shapiro–Wilk normality test, comparisons between the performances of OCD patients and the control group were assessed using the Mann–Whitney *U* rank test and independent *t*-test. Median values and ranges or means and SDs are presented in Results, respectively.

The statistical analysis was performed in TIBCO Statistica 13.4.0.14 (1984-2018 TIBCO Software Inc. USA).

## Results

Each pediatric OCD patient without any comorbidities finished all of the aforementioned tasks, with only two of patients being ineligible for the visual paradigm based on disabilities in color sight, as measured by the Ishihara plates (we let the children do the task so that they did not feel excluded but did not use their results). Apart from this small exclusion and a minor technical error with the multisensory recording, each participant was able to finish all three paradigms and learn all of the associations. Thus, we present data of 29 pediatric OCD patients in the visual paradigm, 31 in the auditory paradigm, and 30 in the multisensory paradigm with their matched healthy controls.

### Comparison of Performances in the Three Paradigms Between OCD Patients and the Control Group

#### Visually Guided Associative Learning Paradigm

**Figure 2:**
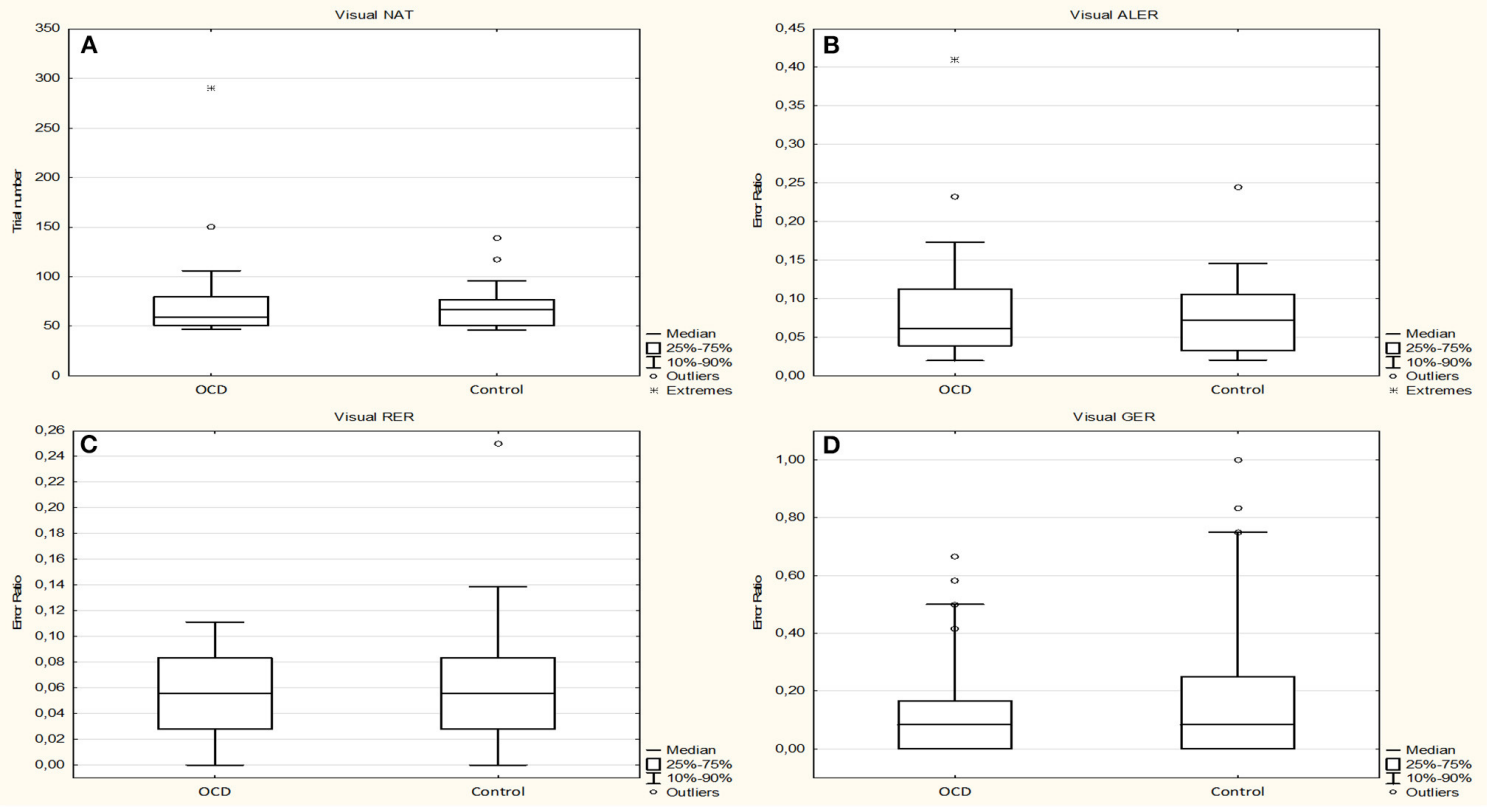
Performance of the obsessive–compulsive disorder (OCD) children in the visually guided equivalence learning paradigm. **(A)** Shows the number of trials necessary to complete the acquisition phase of the paradigm (NAT). **(B)** Shows the error ratios in the acquisition phase(ALER). **(C,D)** Show the error ratios in the two parts of the test phase: retrieval (RER) and generalization (GER), respectively. In each panel, the first plot shows the performance of the patients, and the second plot shows the performance of the control children. The lower margin of the boxes represents the 25th percentile, the square within the boxes marks the median, and the upper margin of the boxes represents the 75th percentile. The whiskers encompass the 10 and 90 percentiles of the data. The points symbolize the outliers.

After the normality tests (the data sets showed no normal distribution except ALER of the control group), we compared the performances with Mann–Whitney *U* tests. None of the investigated performances and reaction times were different between the OCD and healthy children groups. The median of the NAT in the OCD group was 59.0 (range, 44–290; *n* = 29) and 67.0 (range, 42–139; *n* = 29) in the control group (Mann–Whitney rank test *U* = 402, *p* = 0.779). The median of the ALER was 0.0612 (range, 0.00–0.4103; *n* = 29) for the OCD group and 0.0725 (range, 0.00–0.2446; *n* = 29) for the control group (Mann–Whitney rank test *U* = 426, *p* = 0.938). The median of the RER was 0.0556 (range, 0.00–0.1667; *n* = 29) for the OCD group and 0.0556 (range, 0.00–0.25; *n* = 29) for the control group (Mann–Whitney rank test *U* = 404, *p* = 0.8). The median of the GER was 0.0833 (range, 0.00–0.6667; *n* = 29) for the OCD group and 0.0833 (range, 0.00–1.00; *n* = 29) for the control group (Mann–Whitney rank test *U* = 371, *p* = 0.431) (Figure 2).

**Figure 3:**
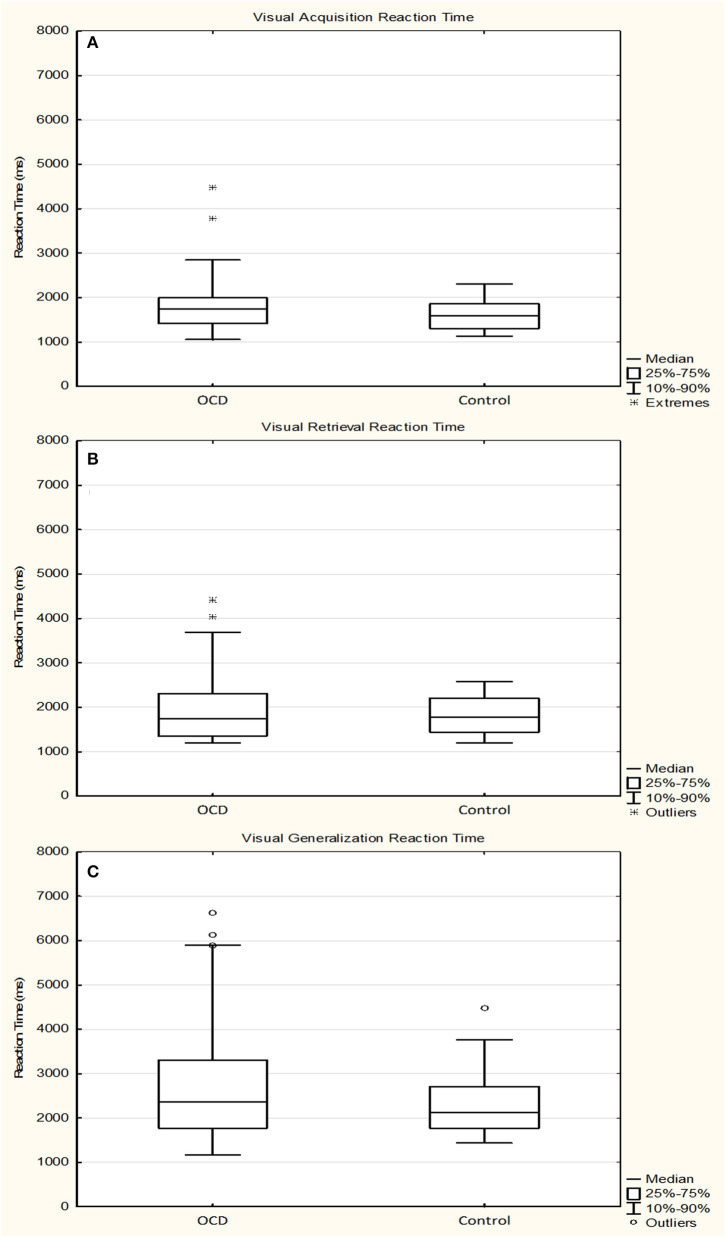
Response times of the obsessive–compulsive disorder (OCD) children in the visually guided equivalence learning paradigm. **(A)** Shows the response times during the acquisition phase, while **(B,C)** Show the response times during the retrieval and generalization parts of the test phase, respectively. The lower margin of the boxes represents the 25th percentile, the square within the boxes marks the median, and the upper margin of the boxes represents the 75th percentile. The whiskers encompass the 10 and 90 percentiles of the data. The points symbolize the outliers. All response times are presented in milliseconds.

The median of the RTs during the acquisition phase was 1,744.9355 ms (range, 967.9091–4470.9149 ms; *n* = 29) for the OCD group and 1,590.3699 ms (range, 1,037.2542–2,455.3953 ms; *n* = 29) for the control group (Mann–Whitney rank test *U* = 472, *p* = 0.428). During the retrieval part of the test phase, the median of the RTs was 1,738.6207 ms (range, 951.5882–4,425.2941; *n* = 29) for the OCD group and 1,785.8387 ms (range, 1,054.6000–2,696.8235 ms; *n* = 29) for the control group (Mann–Whitney rank test *U* = 418, *p* = 0.975). In the generalization part, the median of the RTs was 2,359.8182 ms (range, 839.1111–6,630.1667 ms; *n* = 29) for the OCD group and 2,126.6667 ms (range, 1,102.6667–4,481.0000 ms; *n* = 27) for the control group (Mann–Whitney rank test *U* = 351, *p* = 0.512) (Figure 3).

#### Auditory-Guided Associative Learning Paradigm

The normality test revealed no normal distribution for all of the following data sets in healthy controls and OCD patients. The investigated performances and reaction times were not different (with Mann–Whitney *U* tests) between the OCD and healthy children groups.

**Figure 4:**
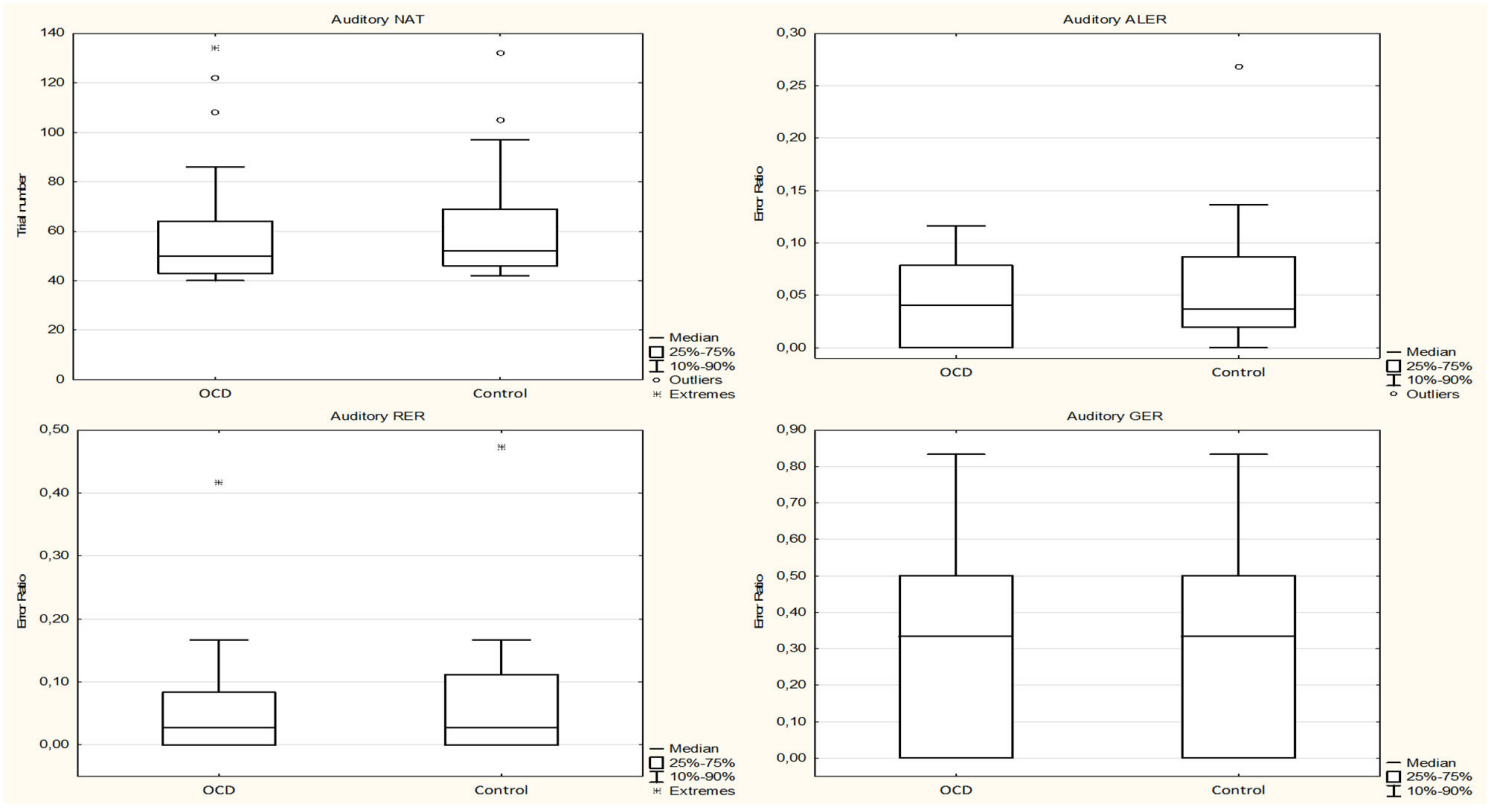
Performance of the obsessive–compulsive disorder (OCD) children in the auditory-guided equivalence learning paradigm. In each panel, the first plot shows the performance of the OCD patients, and the second plot shows the performance of the healthy control group. Other conventions are the same as in Figure 2.

The median of the NAT was 50.0 (range, 39–134; *n* = 31) in the OCD group and 52.0 (range, 40–132; *n* = 31) in the control group (Mann–Whitney rank test *U* = 442, *p* = 0.592). The median of the ALER was 0.0408 (range, 0.00–0.0465; *n* = 31) for the OCD patients and 0.0370 (range, 0.00–0.0577; *n* = 31) for the control group (Mann–Whitney rank test *U* = 444, *p* = 0.611). The median of the RER was 0.0278 (range, 0.00–0.0609; *n* = 31) for the OCD group and 0.0278 (range, 0.00–0.0690; *n* = 31) for the control group (Mann–Whitney rank test *U* = 452, *p* = 0.685). The median of the GER was 0.3333 (range, 0.00–1.00; *n* = 31) for the OCD patients and 0.3333 (range, 0.00–1.00; *n* = 31) for the control group (Mann–Whitney rank test *U* = 474, *p* = 0.937) (Figure 4).

**Figure 5:**
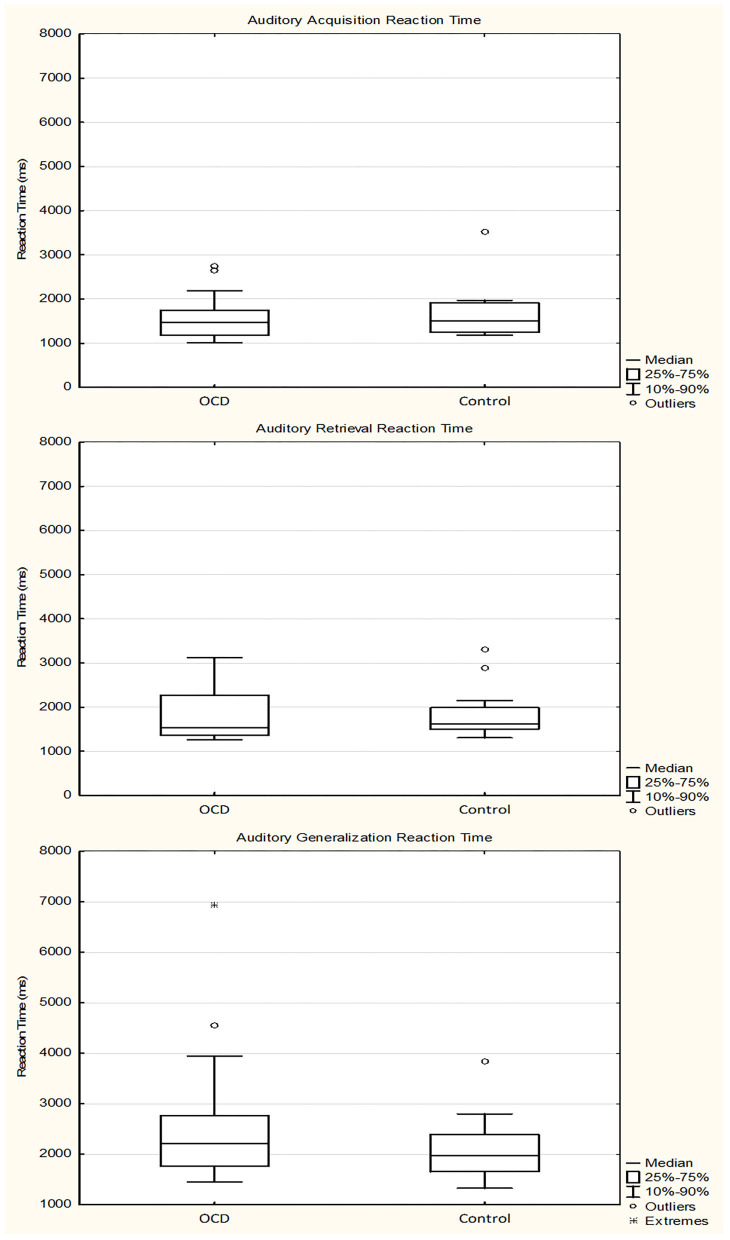
Response times of the obsessive–compulsive disorder (OCD) children in the auditory-guided equivalence learning paradigm. In each panel, the first plot shows the performance of the OCD patients, and the second plot shows the performance of the healthy control group. Other conventions are the same as in Figure 3.

The median of the RTs during the acquisition phase was 1,468.4716 ms (range, 935.7500–2,743.2927 ms; *n* = 31) for the OCD group and 1,499.3214 ms (range, 1,027.7317–3,520.9535 ms; *n* = 31) for the control group (Mann–Whitney rank test *U* = 455, *p* = 0.725). During the retrieval part of the test phase, the median of the RTs was 1,542.1429 ms (range, 1,145.7059–3,436.9375; *n* = 31) for the OCD group and 1,623.2059 ms (range, 1,198.3704–3,296.4000; *n* = 31) for the control group (Mann–Whitney rank test *U* = 470, *p* = 0.888). In the generalization part, the median of the RTs was 2,219.2500 ms (range, 733.5000–6,942.0000 ms; *n* = 29) for the OCD group and 1,976.3222 ms (range, 1,159.7500–3,841.0000 ms; *n* = 30) for the control group (Mann–Whitney rank test *U* = 513, *p* = 0.240) (Figure 5).

#### Multisensory-Guided Associative Learning Paradigm

**Figure 6:**
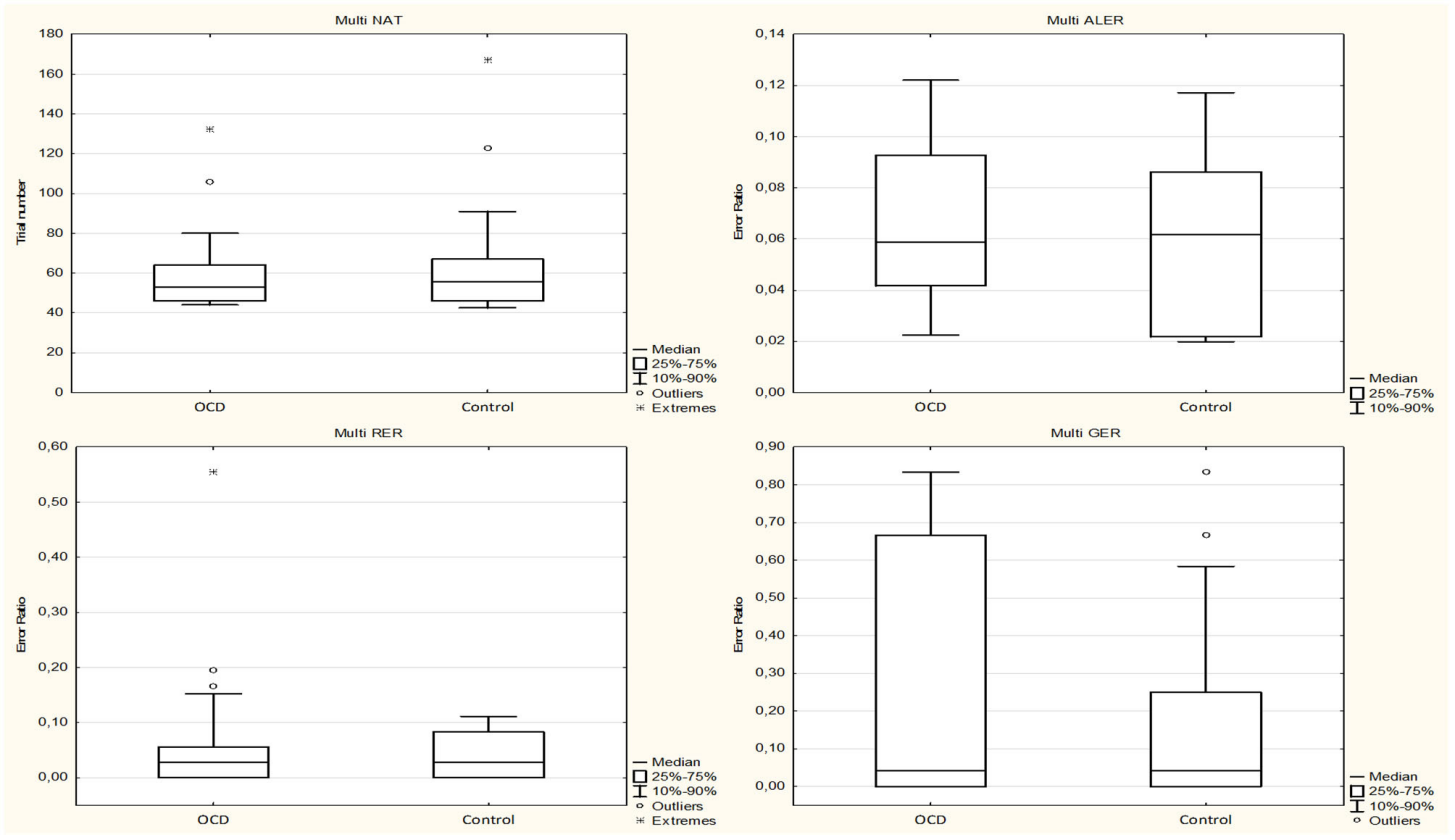
Performance of the obsessive–compulsive disorder (OCD) children in the multisensory-guided equivalence learning paradigm. In each panel, the first plot shows the performance of the OCD patients, and the second plot shows the performance of the healthy control group. Other conventions are the same as in Figure 2.

The normality test revealed no normal distribution for all of the following data sets in healthy controls and OCD patients (the only exception was the ALER of the OCD patient group, which showed normal distribution). None of the investigated performances and reaction times were different (with Mann–Whitney *U* tests) between the OCD and healthy children groups. The median of the NAT was 53.0 (range, 41–132; *n* = 30) in the OCD group and 55.5 (range, 41–167; *n* = 30) in the control group (Mann–Whitney rank test *U* = 423, *p* = 0.695). The median of the ALER was 0.0589 (range, 0.00–0.1509; *n* = 30) for the OCD patients and 0.0617 (range, 0.00–0.1463; *n* = 30) in the control group (Mann–Whitney rank test *U* = 508, *p* = 0.391). The median of the RER was 0.0278 (range, 0.00–0.5556; *n* = 30) for the OCD group and 0.0278 (range, 0.00–0.1944; *n* = 30) for the control group (Mann–Whitney rank test *U* = 422, *p* = 0.672). The median of the GER was 0.0417 (range, 0.00–1.00; *n* = 30) for the OCD patients and 0.0417 (range, 0.00–0.8333; *n* = 30) for the control group (Mann–Whitney rank test *U* = 476, *p* = 0.680) (Figure 6).

**Figure 7:**
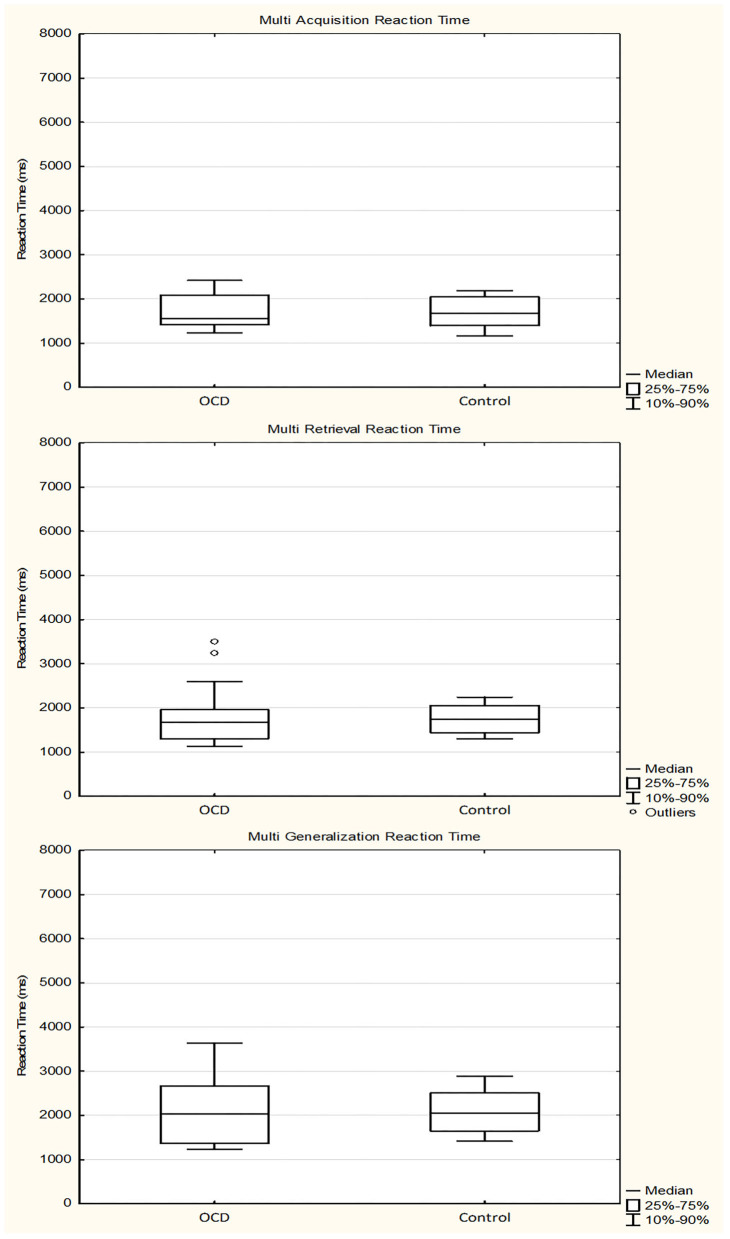
Response times of the obsessive–compulsive disorder (OCD) children in the multisensory guided equivalence learning paradigm. In each panel, the first plot shows the performance of the OCD patients, and the second plot shows the performance of the healthy control group. Other conventions are the same as in Figure 3.

The median of the RTs during the acquisition phase was 1,565.0556 ms (range, 1,085.8824–2,796.3898 ms; *n* = 30) for the OCD group and 1,677.4331 ms (range, 1,076.4130–2,595.6279 ms; *n* = 30) for the control group (Mann–Whitney rank test *U* = 454, *p* = 0.959). During the retrieval part of the test phase, the median of the RTs was 1,672.5071 ms (range, 843.5333–3,502.8667 ms; *n* = 30) for the OCD group and 1,739.1429 ms (range, 1,246.6857–2,354.7353 ms; *n* = 30) for the control group (Mann–Whitney rank test *U* = 427, *p* = 0.739). In the generalization part, the median of the RTs was 2,031.8333 ms (range, 1,060.7500–4,381.1667 ms; *n* = 29) for the OCD group and 2,055.4167 ms (range, 1,241.4167–3,742.3000 ms; *n* = 30) for the control group (Mann–Whitney rank test *U* = 417, *p* = 0.791) (Figure 7).

### Comparison of Performances in the Three Paradigms Between Groups of Medicated and Unmedicated Patients With OCD

In order to get information whether the medication could influence the performances of the OCD patients, we have compared the performances of the two patients (medicated and unmedicated) and their two matched healthy control subgroups in a quadruple multiple comparison with Kruskal–Wallis ANOVA test. These results revealed no significant differences (*p* > 0.05) among the four subgroups in any of the investigated metrics (NAT, ALER, RER, and GER) and in any of the paradigms (visual, auditory, and multisensory). Because of the absence of significant differences, we present the detailed pairwise comparison between medicated and unmedicated pediatric OCD patients only.

#### Visual Paradigm

The normality testing revealed normal distribution of the ALER and GER of the unmedicated groups and the RER of both groups. None of the tested parameters differed between the two groups in the visual task.

The median of the NAT in the medicated group was 59.0 (range, 47–290; *n* = 15) and 59.0 (range, 44–102; *n* = 14) in the unmedicated group (Mann–Whitney rank test *U* = 123, *p* = 0.445). The median of the ALER was 0.0769 (range, 0.00–0.4103; *n* = 15) for the medicated group and 0.0609 (range, 0.0196–0.2323; *n* = 14) for the unmedicated group (Mann–Whitney rank test *U* = 96.5, *p* = 0.727). The median of the RER was 0.0556 (range, 0.00–0.1667; *n* = 15) for the medicated group and 0.0417 (range, 0.00–0.1389; *n* = 14) for the unmedicated group [independent samples *t*-test *t*(27) = 0.122, *p* = 0.904].

The median of the GER was 0.00 (range, 0.00–0.6667; *n* = 15) for the medicated group and 0.0833 (range, 0.00–0.5833; *n* = 14) for the unmedicated group (Mann–Whitney rank test *U* = 87, *p* = 0.425).

#### Auditory Paradigm

After normality testing, only the ALER of the unmedicated group and the GER of the medicated group showed normal distribution. None of the tested parameters differed significantly between the two groups in the auditory task.

The median of the NAT was 54.5 (range, 39–108; *n* = 16) in the medicated group and 49.0 (range, 39–134; *n* = 15) in the unmedicated group (Mann–Whitney rank test *U* = 116, *p* = 0.890). The median of the ALER was 0.0305 (range, 0.00–0.1296; *n* = 16) for medicated group and 0.0465 (range, 0.00–0.1343; *n* = 15) for the unmedicated group (Mann–Whitney rank test *U* = 103, *p* = 0.511). The median of the RER was 0.0417 (range, 0.00–0.4167; *n* = 16) for the medicated group and 0.0278 (range, 0.00–0.1111; *n* = 15) for the unmedicated group (Mann–Whitney rank test *U* = 154, *p* = 0.177). The median of the GER was 0.375 (range, 0.00–1.00; *n* = 16) for medicated patients and 0.0833 (range, 0.00–1.00; *n* = 15) for the unmedicated ones (Mann–Whitney rank test *U* = 126, *p* = 0.809).

#### Multisensory Paradigm

Normality testing has yielded normal distribution of ALER in both medicated and unmedicated groups. None of the tested parameters significantly differed between the medicated and unmedicated groups in the multisensory task.

The median of the NAT was 52.0 (range, 41–132; *n* = 15) in the medicated group and 54.0 (range, 44–106; *n* = 15) in the unmedicated group (Mann–Whitney rank test *U* = 98, *p* = 0.561). The median of the ALER was 0.0588 (range, 0.00–0.1304; *n* = 15) for the medicated patients and 0.0588 (range, 0.0222–0.1509; *n* = 15) for the unmedicated group [independent samples *t*-test *t*(28) = −0.775, *p* = 0.445].

The median of the RER was 0.00 (range, 0.00–0.5556; *n* = 15) for the medicated group and 0.0278 (range, 0.00–0.1944; *n* = 15) for the unmedicated group (Mann–Whitney rank test *U* = 95, *p* = 0.458). The median of the GER was 0.00 (range, 0.00–0.8333; *n* = 15) for the medicated group and 0.0833 (range, 0.00–1.00; *n* = 15) for the unmedicated group (Mann–Whitney rank test *U* = 91, *p* = 0.350).

None of the RTs differed significantly between the medicated and unmedicated or patient subgroups and their matched control groups.

We also compared the abovementioned performance metrics (NAT, ALER, RER, GER, and RTs) between the medicated and unmedicated OCD children and their respective controls. Furthermore, we compared the performance of the two control groups (medicated and unmedicated) and did not find any significant difference between them. Since none of the tests showed any significant difference, we do not provide a detailed account of these comparisons.

## Discussion

To our knowledge, the present study is the first that has addressed visual-, auditory-, and multisensory (audiovisual)-guided acquired equivalence learning in children and adolescents suffering from OCD. The original visually guided Rutgers Acquired Equivalence Test [the other name is the face–fish test (8)] was developed in order to learn about visually guided associative learning of neurological patients with basal ganglia and hippocampus dysfunction. A great advantage of this test is that each phase of the paradigm has well-described neural substrates (12, 25). The test can be divided into two parts. The first is the acquisition phase, where the subjects have to learn particular visual stimuli combinations based on the feedback of a computer program. The building of associations of new stimuli is predominated by the function of the basal ganglia (26, 27), and the coding and restoration of associations are connected to the frontal and medial temporal lobe function (28). The second part of the applied behavioral learning paradigm is the test phase (retrieval and generalization), which is dominated by the hippocampus–MT lobe system, and the contribution of the basal ganglia to this test phase is much weaker (25, 28). The test was applied later for other neurological and psychiatric disorders, i.e., schizophrenia, migraine, and Alzheimer's disease (9, 11–14) and in healthy subjects (10, 29). It is known from earlier studies that both the basal ganglia and the hippocampi, which are critically involved in acquired equivalence learning, process not only visual but also auditory and multisensory information (16–19). However, multisensory-guided acquired equivalence learning had not yet been investigated. Heaving realized the absence of any auditory- or multisensory-guided acquired equivalence learning tests, we developed and then validated an auditory and multisensory test in healthy humans (20). Applying these three tests (visual-, auditory-, and multisensory-guided acquired equivalence tests), we investigated the associative learning functions of children and adolescents suffering from OCD.

The remaining sensory-guided equivalence learning, independent from the modality of the stimuli, is an interesting finding because the majority of earlier studies showed that cognitive functions were altered in these patients (i.e., implicit sequence learning (30), spatial attention (31), and nonverbal memory (32) and, only in rare cases, did not find significant impairments in cognitive functions). Because of the strong involvement of the frontal cortex–basal ganglia loops and the hippocampi in the pathogenesis of OCD, the hypothesis of the present study was that the performance in these paradigms would be affected. However, the OCD patients could build, recall, and generalize the associations with the same effectiveness as the healthy control children matched for sex, age, and IQ level. The possible explanations for this unaffected learning function is that the structural differences observed in childhood (3, 4) and MRI studies revealed that the volume reduction in the hippocampi, which was observed in adult OCD patients, was not detectable in children with OCD [for a meta-analysis, see (33)] and do not even functionally appear in childhood. Another possible explanation is that OCD primarily affects the ventral (limbic) but does not affect or has a much weaker impact on dorsal corticostriatal loops (5, 34). Similarly to stimulus response or habit learning (26, 35), acquired equivalence learning, which is primarily connected to the dorsal frontostriatal loops (26), was not significantly altered in children with OCD.

Similarly to the remaining acquisition function, the retrieval and generalization parts of the test phase, which primarily depend upon the function of the hippocampus–mediotemporal lobe—a system that is less involved in the pathogenesis of OCD (33) than the basal ganglia–frontal cortex loops—were not altered either. Recent results have described some morphological changes in the hippocampi of adult OCD patients, but this has not yet been described in children and adolescents with the same disorder (36–40).

The question is raised as to whether the same performances in the psychophysical results were not due to the longer response times of the OCD patients. One argument could be that the longer response times of the OCD patients resulting from their compulsions could enhance their performances and decrease the number of bad decisions. However, similarly to the performances in the psychophysical tests, there were no differences between the response times of the OCD patients and the healthy control children. Thus, the participants with OCD took no more time to make decisions than the healthy controls.

We have to mention here that only patients with severe or moderate OCD symptoms get into the Vadaskert Child and Adolescent Hospital (Budapest, Hungary) patient care system, so they participated in our study. Patients with severe symptoms are taking medication, and those with moderate symptoms receive only psychotherapy in this system. To exclude the effect of medication and the severity on the performances of the OCD patients, we have compared the performances of the medicated and unmedicated OCD patient subgroups. These comparisons revealed absolutely no differences, which suggest that the medication could not significantly affect the metrics (NAT, ALER, RER, and GER) in any of the applied paradigms (visual, auditory, and multisensory).

In summary, the children suffering from OCD had the same performance as the controls in all phases of the applied visual-, auditory-, and multisensory-guided associative learning paradigms. Thus, both the acquisition and the test parts, which are primarily connected to the function of the basal ganglia and the hippocampi, respectively, were not negatively affected by children suffering in OCD. Our results support the findings that the structural changes in basal ganglia and hippocampi detected in adult OCD patients are not as pronounced in children (35), which could be the explanation of the maintained associative equivalence learning functions in children suffering from OCD.

## Data Availability Statement

The raw data supporting the conclusions of this article will be made available by the authors, without undue reservation.

## Ethics Statement

The studies involving human participants were reviewed and approved by Ministry of Human Capacities, Budapest, Hungary (11818-6/2017/EÜIG). Written informed consent to participate in this study was provided by the participants' legal guardian/next of kin.

## Author Contributions

ÁPe: formal analysis, investigation, data curation, writing - original draft, and visualization. GE: conceptualization, methodology, investigation, writing - original draft, and project administration. LN: investigation and project administration. OH and DÖ: investigation. APu: software and investigation. AN: conceptualization, methodology, resources, writing - original draft, supervision, project administration, and funding acquisition. PN: investigation and resources. SK: conceptualization and methodology. All authors contributed to the article and approved the submitted version.

## Conflict of Interest

The authors declare that the research was conducted in the absence of any commercial or financial relationships that could be construed as a potential conflict of interest.
