# Supplementary material for: Maintained Visual-, Auditory-, and Multisensory-Guided Associative Learning Functions in Children With Obsessive–Compulsive Disorder
Source: Front Psychiatry. 2020 Nov 26;11:571053. doi: 10.3389/fpsyt.2020.571053 (PMC7726134; doi:10.3389/fpsyt.2020.571053)
Supplement: Supplementary file 1 [file Data_Sheet_1.pdf]

Children with OCD / Medicated vs matched control group:

Modality	Variable	Statistical test	Singnificance
Visual	NAT	Mann-Whitney rank test: $U = 120$,	$p = 0.787$
	ALER	Mann-Whitney rank test: $U = 106$	$p = 0.819$
	RER	independent samples t-test: $t(28) = -0.101$	$p = 0.920$
	GER	Mann-Whitney rank test: $U = 89.5$	$p = 0.328$
Auditory	NAT	Mann-Whitney rank test: $U = 114$	$p = 0.610$
	ALER	Mann-Whitney rank test: $U = 108$	$p = 0.459$
	RER	Mann-Whitney rank test: $U = 130$	$p = 0.954$
	GER	independent samples t-test: $t(30) = -0.0495$	$p = 0.961$
Multisensory	NAT	Mann-Whitney rank test: $U = 97$	$p = 0.533$
	ALER	independent samples t-test: $t(28) = 0.0301$	$p = 0.976$
	RER	Mann-Whitney rank test: $U = 84.5$	$p = 0.236$
	GER	Mann-Whitney rank test: $U = 103$	$p = 0.685$

Children with OCD / Unmedicated vs matched control group:

Modality	Variable	Statistical test	Singnificance
Visual	NAT	Mann-Whitney rank test: $U = 80.5$	$p = 0.434$
	ALER	Mann-Whitney rank test: $U = 110$	$p = 0.613$
	RER	Mann-Whitney rank test: $U = 88.5$	$p = 0.672$
	GER	Mann-Whitney rank test: $U = 96$	$p = 0.944$
Auditory	NAT	Mann-Whitney rank test: $U = 114$	$p = 0.950$
	ALER	Mann-Whitney rank test: $U = 112$	$p = 0.983$
	RER	Mann-Whitney rank test: $U = 105$	$p = 0.761$
	GER	Mann-Whitney rank test: $U = 10$	$p = 0.866$
Multisensory	NAT	Mann-Whitney rank test: $U = 114$	$p = 0.983$
	ALER	Mann-Whitney rank test: $U = 140$	$p = 0.271$
	RER	Mann-Whitney rank test: $U = 130$	$p = 0.448$
	GER	Mann-Whitney rank test: $U = 134$	$p = 0.345$
